# Littoral cell angioma of a huge spleen with peripheral blood pancytopenia in a 14-year-old boy: a case report and review of the literature

**DOI:** 10.3389/fped.2024.1473930

**Published:** 2024-10-31

**Authors:** Tianyu Gao, Xingang Wang, Qiuya Wei, Chen Wang, Yong Fan, Yuebin Wang

**Affiliations:** Department of General Surgery, The Second Hospital of Lanzhou University, Lanzhou, China

**Keywords:** splenic tumor, case report, children, vascular neoplasm, littoral cell angioma

## Abstract

**Background:**

Splenic littoral cell angioma (LCA) is an exceptionally uncommon malignant potential vascular tumor with infrequent occurrences in pediatric patients. Due to its reliance on histopathological analysis for diagnosis, LCA may be mistakenly identified as other splenic tumors. Patients with LCA may experience anemia or thrombocytopenia, but peripheral blood pancytopenia is infrequent.

**Case report:**

A 14-year-old boy presented with peripheral blood pancytopenia, necessitating hospitalization after splenomegaly was identified during a physical examination. Following the exclusion of hematological disorders, a splenectomy was conducted; histopathological examination confirmed the diagnoses of LCA. No metastases or recurrences were observed during the 8-month follow-up. To the best of our knowledge, this case represents the first instance of LCA associated with pancytopenia in a pediatric patient.

**Conclusion:**

LCA can lead to iron-deficiency anemia or thrombocytopenia, with rare occurrences of pancytopenia, potentially resulting in misdiagnosis as a hematological disorder. Surgical intervention remains an effective treatment for LCA.

## 1 Introduction

In splenic tumors, lymphoma is the most common, while other primary and secondary neoplasms are rare (1). Primary splenic tumors in children are even rarer, and most are benign tumors, accounting for 0.03% of all tumors (2). Most splenic tumors can be diagnosed with the help of imaging tools, assisting in judging the timing for surgery (3).

Littoral cell angioma (LCA) is extremely rare among splenic tumors, which most commonly occurs in middle-aged adults, with only 15 reported cases in minors, and is even rarer in children (4). LCA patients usually present with splenomegaly and other conditions, such as other tumors and hematologic disorders, but LCA with peripheral blood pancytopenia is uncommon (5). This case report introduces a 14-year-old boy with LCA and peripheral blood pancytopenia, and the clinical characterization, imaging features, and pathological features of LCA in children are summarized by a literature review to provide meaningful treatment recommendations for clinicians.

## 2 Case report

A 14-year-old boy was admitted to the General Surgery Department of the local hospital of Lanzhou University for diarrhea, who had growth retardation and was only 148.0 cm tall (range: 152.3–179.4 cm). Upon abdominal examination, the abdomen appeared slightly bulged, with the firm spleen easily palpable, indication grade III splenomegaly, while the liver was not palpable.

**Figure 1:**
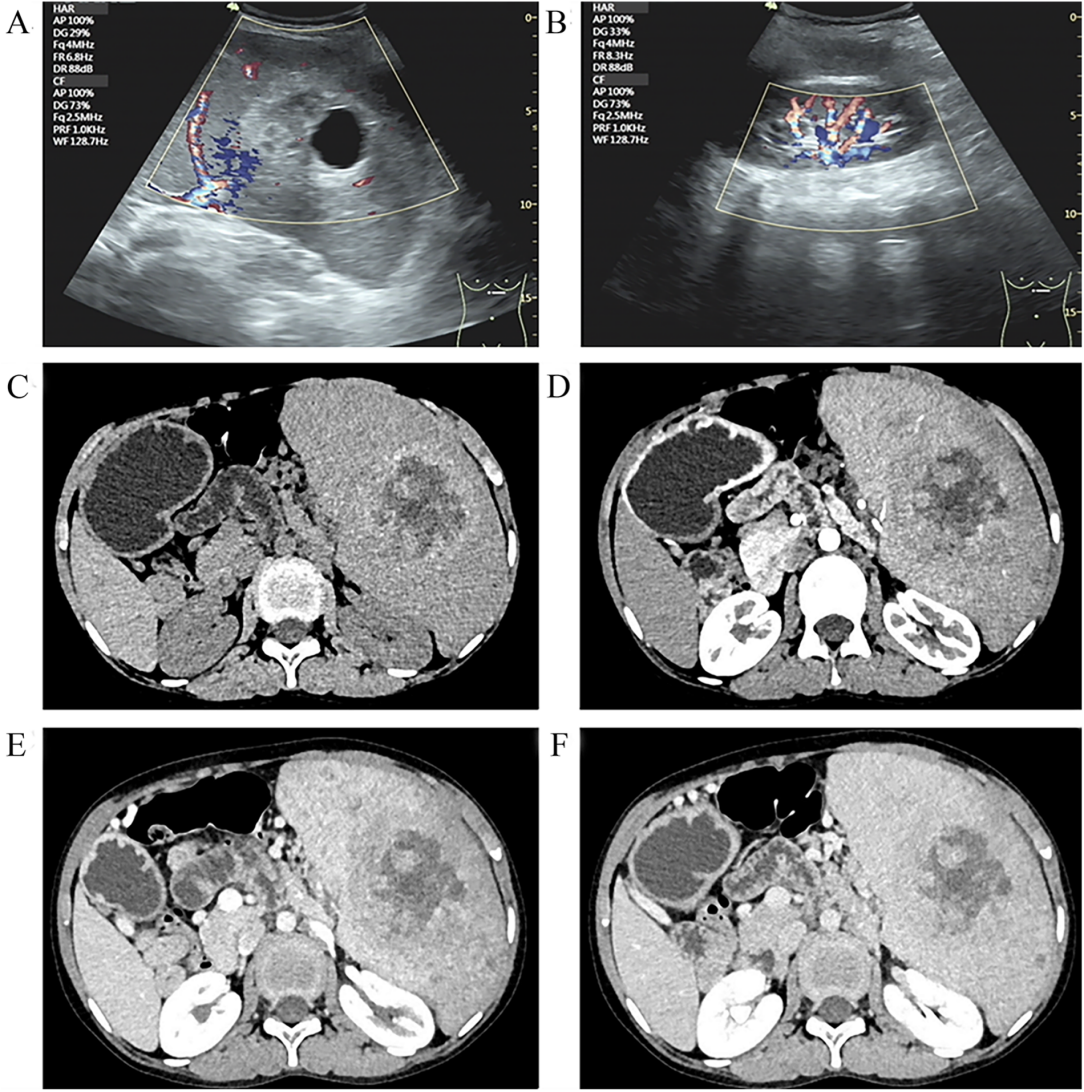
Ultrasound images of LCA **(A,B)**: ultrasound indicated that the internal echoes of the spleen were heterogeneous, measuring approximately 7.4 cm × 6.8 cm. CT images of LCA **(C–F)** indicated multiple low-density lesions: **(C)** Non-contrast-enhanced CT showed low-density splenic masses; **(D)** the arterial phase of contrast-enhanced CT became clearer, though the peripheral enhancement of the tumor remained unobvious; **(E)** the venous phase of contrast-enhanced CT showed the enhancement degree of the tumor mimicking normal spleen tissue; and **(F)** the delayed phase of contrast-enhanced CT showed the highest CT value.

Laboratory test results are as follows: white blood cells 2.27 × 10^9^/L (range: 4.10–11.00 × 10^9^/L), neutrophils 0.88 × 10^9^/L (range: 1.80–8.30 × 10^9^/L), lymphocytes 1.18 × 10^9^/L (range: 1.20–3.80 × 10^9^/L), red blood cells 3.83 × 10^12^/L (range: 4.50–5.90 × 10^12^/L), platelets 40 × 10^9^/L (range: 150–407 × 10^9^/L), and hemoglobin 109 g/L (range: 129–172 g/L). Hepatic function test results were abnormal: total bilirubin 45.8 μmol/L (range: <26.0 μmol/L), direct bilirubin 10.2 μmol/L (range: <4.0 μmol/L), and indirect bilirubin 35.6 μmol/L (range: 0.0–22.1 μmol/L). The tumor markers were normal. Additional preoperative detailed conditions of the patient are organized in Supplementary Table S1. To rule out hematological disorders, we performed a bone marrow aspiration, which showed an increase in megakaryocytic and erythrocytic series but a decrease in granulocytic series (Supplementary Figure S1). In addition, we also carried out an analysis of genetic testing, and the results were normal, allowing us to rule out aplastic anemia and leukemia. Abdominal ultrasonographic examination revealed that the thickness of the splenic hilum was about 8.7 cm, the spleen index was 83 cm^2^, and the lower margin of spleen extended 5.3 cm below ribs, the internal echoes of the spleen were heterogeneous, measuring about 7.4 cm × 6.8 cm, and the blood flow signal was punctate, suggesting splenic hemangioma (Figure 1). To further determine the size of the spleen and tumor distribution, imageology detection was performed. Enhanced computed tomography (CT) of the abdomen showed an enlarged, lumpy spleen with mixed density and dense, multiple collateral circulations, raising the possibility of splenic malignancy; also, the enhancement signal was not clear (Figure 1). After assessing the patient's condition, we decided to proceed with surgery. Because of the patient's huge spleen, a laparotomic splenectomy was performed. Further intraoperative details concerning the patient's conditions are compiled in Supplementary Table S2.

**Figure 2:**
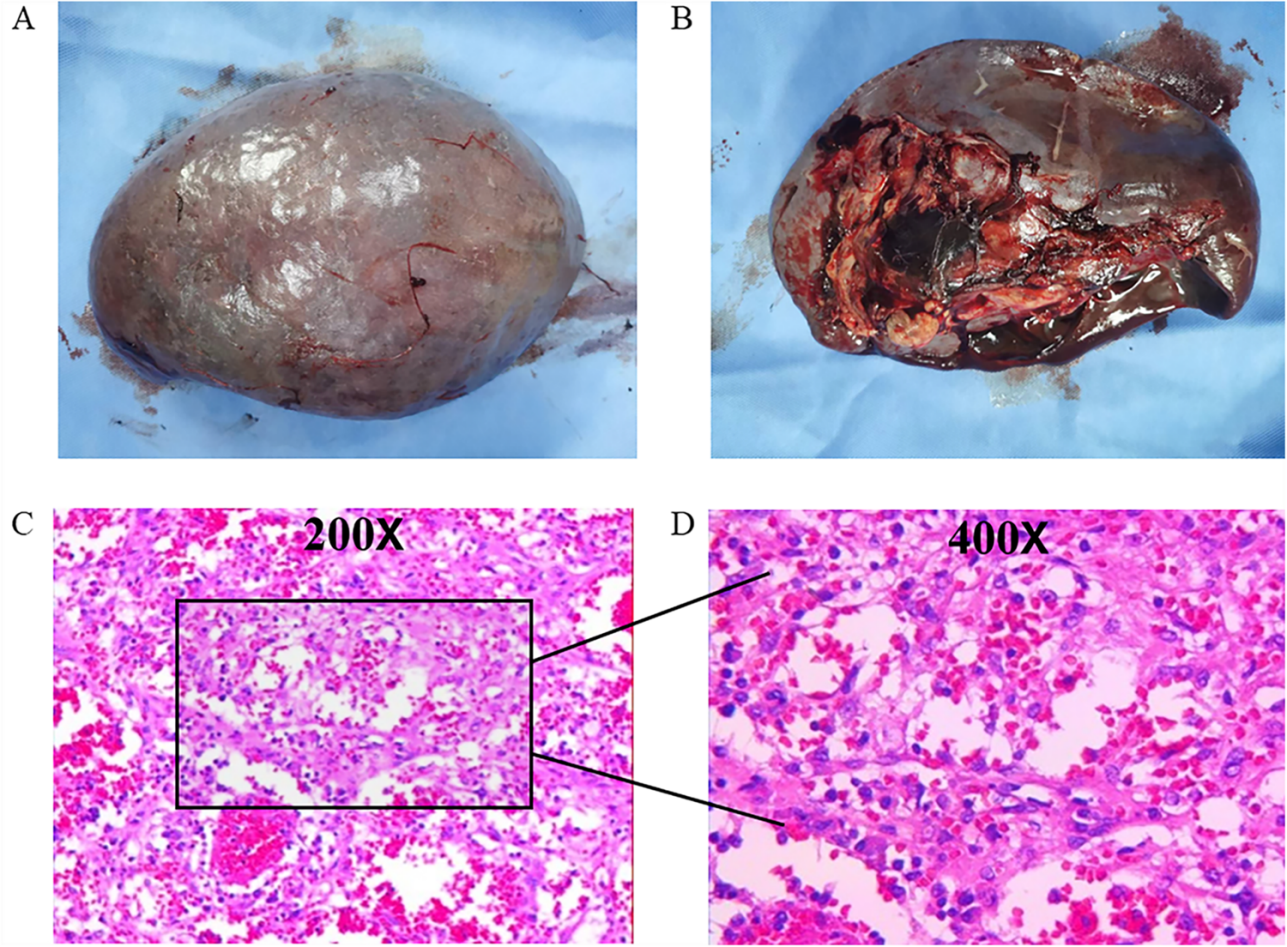
Pathological features of LCA: **(A)** the spleen measured 17.5 cm × 14 cm × 8.5 cm; **(B)** multiple gray–yellow nodules on the cut surface; and **(C,D)** the tumor consisted of vascular lumens of varying sizes, lined with blood vessels and a single layer of proliferating endothelial cells.

**Figure 3:**
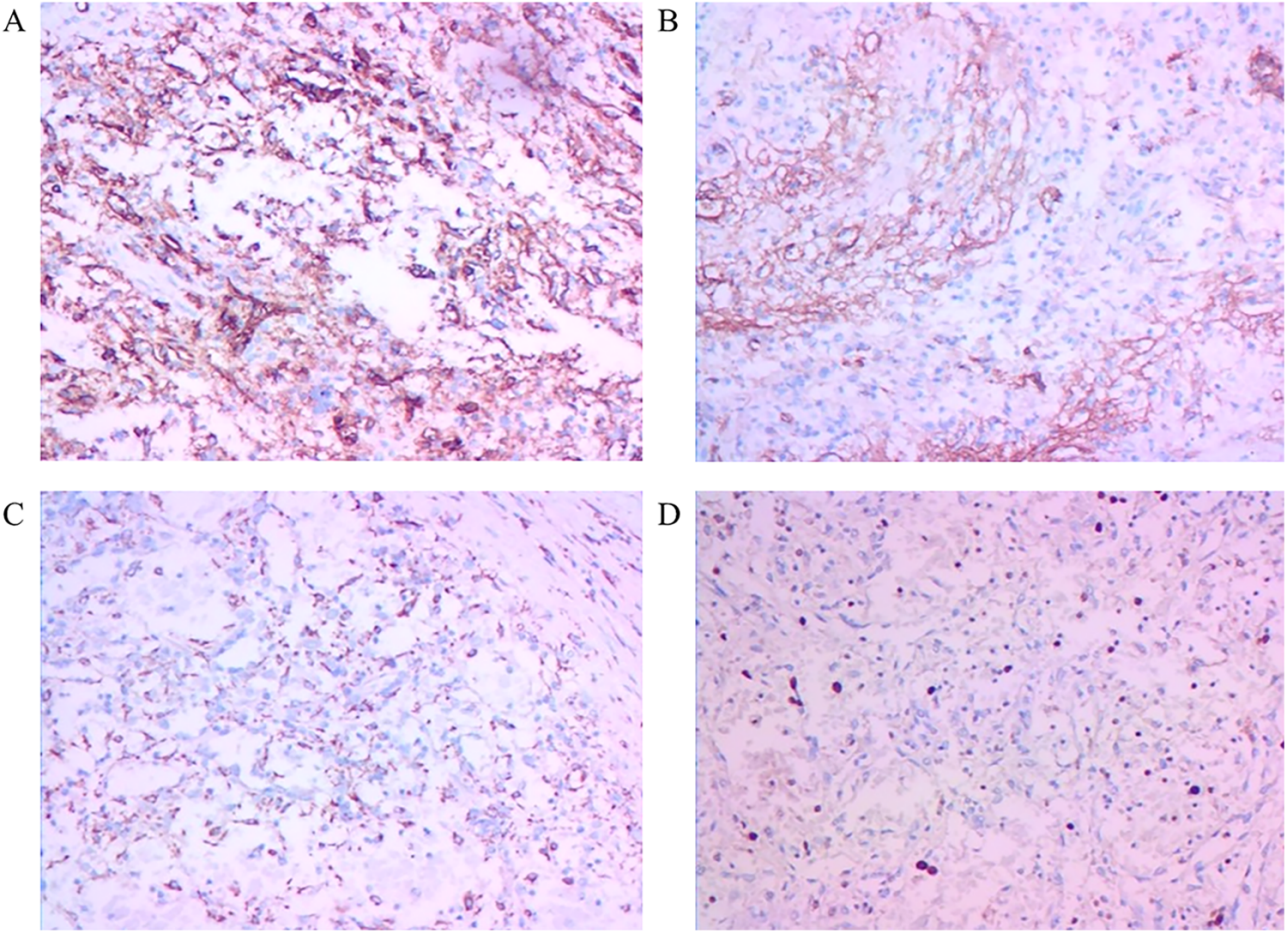
Immunohistochemistry features of LCA: **(A)** CD31(+) staining, **(B)** CD34(+) staining, **(C)** CD68(+) staining, and **(D)** Ki67 (1%) staining (IHC × 200).

Surgical specimens from the spleen revealed that it measured 17.5 cm × 14cm × 8.5 cm, with multiple gray–yellow nodules seen on the cut surface, with the largest measuring 5 cm in diameter (Figure 2). Immunohistochemical (IHC) staining suggested that the tumor tissue was seen in the red pulp, anastomosing each other into a network of blood vessels, and the center of the blood vessels exhibited papillary hyperplasia, surrounded by several infarcts. Tumor cells were positive for CD31. CD34, CD68, and Ki67 (1%) but negative for CD8 and CKp (Figure 3). These tests confirmed that the child had LCA, and the patient's blood indexes gradually returned to normal after the operation. During an 8-month follow-up after the end of treatment, the tumor did not relapse or metastasize.

## 3 Discussion

LCA is a rare splenic tumor that mainly occurs in the adults, with the age of onset being about 50 years old (6). The prevalence of LCA in children is very low, with only 12 cases reported. We conducted a thorough analysis of the clinical presentations, imaging characteristics, and pathology results of these cases (Table 1). Further, we contrasted them with the similarities and differences found in our case, providing a more comprehensive understanding of LCA's manifestations and clinical significance in pediatric patients (Supplementary Table S3).

**Table 1:** Clinicopathological and pathological features of LCA in children.

Variables	Total
Number	13
Age	
<4	5
4–8	1
≥8	7
Gender	
Female	5
Male	8
Clinical manifestations	
Splenomegaly	6
Abdominal symptoms	7
Asymptomatic	2
Fever	1
Purpura	1
Anemia	1
Vomiting	1
Number of tumors	
Multiple	7
Solitary	5
Unknown	1
Year of diagnosis	
<2012	3
≥2012	10
Ultrasonography characteristics	
Strong echoes	1
Low echoes	3
Heterogeneous echoes	2
Unknown	7
CT characteristics	
Low density	4
Mixed density of the spleen	2
Unknown	7
MRI characteristics	
T1 hypointense	3
Equal/slightly prolonged T1 and T2	2
Unknown	8
Routine blood test	
Low WBC	1
Low RBC	5
Low PLT	4
Low fibrinogen	1
Pancytopenia	1
Normal	3
Unknown	3
Histiocytic markers	
CD163	1
CD68	7
Unknown	4
Endothelial markers	
CD31	8
Factor VIII	2
ERG	3
CD34	6
Unknown	4
Other indicators with identification	
CD21	1
EMA	1
Vimentin	2
Ki67	6
Unknown	4
Treatment	
Splenectomy	11
Conservative	1
Unknown	1
Recurrence	
No	6
Unknown	7

WBC, white blood cell; RBC, red blood cell; PLT, platelet; CT, computed tomography; MRI, magnetic resonance imaging.

Patients with LCA are usually found due to abdominal symptoms or by chance, with a similar prevalence rate between males and females, and the condition usually turns out to be multiple lesions in the spleen (2, 7, 8). In the case of children, single and multiple lesions were roughly equal (single lesions: five cases; multiple lesions: seven cases). Although LCA is usually a benign tumor, three cases of malignant LCA have been reported, with one case developing liver metastasis (6, 9–11). No metastases or recurrences were found in any of the pediatric patients, but long-term follow-up and physical examination should be performed after splenectomy. Because the imaging features of other splenic tumors are similar to those of LCA, the most accurate way to diagnose LCA is through a histopathological examination (12). LCA is sometimes positive for transferrin receptors compared with other hemangiomas, which also shows positive staining for endothelial cells and tissue cells, such as factor VIII(+), CD31(+), CD163(+), CD68(+), and CD21(+) (13). Studies have reported that LCA with CD34(+) has the potential to be malignant (14), and nearly half of the cases in children were CD34(+), which indicated that children with LCA should be monitored for long-term postoperative follow-up. There were no recurrences or metastases in any of the children. Formin homology domain-1 antigen expression and double antibody positivity are unique features of LCA, which are also the key markers for distinguishing other diseases (5, 15).

The cause of LCA is still unclear, and preliminary studies suggest that it may be associated with immune system disorders and other visceral tumors. Some studies reported that about 60% of LCA patients have other visceral tumors, and about 12.2% of cases have immune system problems (4, 16). However, some studies showed that the relationship between LCA and splanchnic tumors is vague (17–19), and the specific relationship between the two needs to be further explored. Because cancers rarely occur in childhood (20), no other tumors have been found in pediatric cases of LCA; however, the incidence of LCA increases in children with immunodeficiency (21). Interestingly, LCA may be heritable and familial. Kranzfelder et al. revealed a case where twins developed LCA simultaneously (10). Due to the lack of basic research and large sample analyses, it is impossible to elucidate this situation from genetic susceptibility, and it may be a coincidence. However, we still recommend that patients with a family history of LCA undergo genetic testing.

It is difficult to diagnose LCA only by imaging examinations, although it still has its characteristics. While ultrasonography is relatively simple, its accuracy is low for LCA (22). CT examinations can determine the number of tumors, blood supply, abnormal enhancement, and tumor invasiveness, which can identify most of the characteristics of LCA. CT scans typically reveal low-density splenic masses with delayed enhancement, a feature observed in 10 children. In the case we reported, ringlike enhancement was not obvious, and multiple low-density lesions were discovered in the arterial phase of the enhanced scan; the delayed phase showed the highest CT value, conforming to the typical CT imaging features of LCA. Shen et al. found that for multiple LCA lesions, the number of lesions showed a dynamic change process of “less-more-less” in the enhanced scans (22). Because littoral cells contain hemosiderin, MRI can show the lesion through a spotting sign, characterized by many speckled low-density shadows in the lesions. In almost all of the cases in children, this spotting sign was present. The MRI specificity of LCA is a high signal on T2WI, which is due to hemosiderin stored in the littoral cells. In the young patient group, this characteristic accords with completely.

LCA is very similar to other splenic vascular tumors, making it difficult for surgeons to make accurate judgments based on imaging alone; low-grade angiosarcoma can sometimes mimic LCA. Preoperative fine needle aspiration can help differentiate LCA from other vascular tumors in inoperable patients. The cytological features of LCA in three adult cases revealed a low nuclear-to-cytoplasmic ratio in foamy tumoral cells, with cytoplasm containing hemosiderin (23–26). Anbardar et al. found the same cytological characteristics in the case of an 11-year-old boy (27). It is important to note that intracellular hemosiderin is not a specific diagnostic feature of LCA fine needle aspiration. A surgeon's treatment for LCA is essentially the splenectomy, although chemotherapy may also be effective in patients with suspected LCA distant metastasis (28). Some researchers have suggested that for patients with a solitary lesion, partial splenectomy may be considered to preserve splenic function and prevent the occurrence of life-threatening infections following total splenectomy (21). Laparoscopic splenectomy is recommended for pediatric patients because of its advantages of minimal invasiveness and safety (29). In this case, we chose not to perform laparoscopic splenectomy because of the large size of the spleen. The choice between total splenectomy and partial splenectomy may depend on the size and number of lesions. However, total splenectomy is recommended for patients with a spleen weighing more than 1,500 g, a diameter longer than 20 cm, or who test positive for CD34, because of the greater risk of LCA becoming cancerous (14, 30). Among the pediatric cases, 10 underwent splenectomy, 1 had partial splenectomy, and 1 received conservative treatment.

Approximately 69.7% of LCA patients had splenomegaly, while 46.2% (6/13) of pediatric LCA patients presented with splenomegaly (4). Splenomegaly and associated tumors may lead to a reduction in red blood cells, white blood cells, and platelets, subsequently resulting in symptoms such as anemia, an increased risk of infection, and a propensity for bleeding, which is particularly critical in children, as their immune systems and hematopoietic abilities are not yet fully developed, rendering them more susceptible to such effects (31–33). Taking into account that this pediatric LCA case is afflicted with pancytopenia, we conducted a comprehensive assessment of the child's pancytopenia prior to surgery, encompassing the thorough medical history, physical examination, and laboratory tests, and the necessity for blood transfusion was determined by factors such as the child's age, preoperative hemoglobin levels, volume of surgical blood loss, and cardiovascular response. Further, prophylactic antibiotics were administered 30–60 min prior to the surgery to mitigate the risk of postoperative infection. During the surgical procedure, we closely monitored the child's vital signs and blood parameters, administering fluids and blood transfusions as necessary to address potential hemorrhage and hypovolemia. Postoperatively, we continued monitoring hematological parameters to promptly identify and address complications such as hemorrhage or infection, ensuring a smooth recovery process for this child. Therefore, for pediatric LCA patients with pancytopenia, we should enhance preoperative evaluation, blood transfusion management, preoperative preparation, intraoperative monitoring, and postoperative care to expedite their recovery (34, 35).

LCA is a rare splenic tumor that typically presents clinically as asymptomatic or with vague symptoms such as abdominal pain, splenomegaly, thrombocytopenia, and anemia (4). Consequently, growth issues may exacerbate these symptoms or lead to a delay in diagnosis, as they may be associated with other underlying health issues, thereby affecting the patient's overall health status. Early intervention services can help children receive the support and therapies needed to improve their outcomes (36, 37). In this case, the pediatric patient presented with developmental delays and malnutrition, compounded by communication barriers with the parents, which have contributed to the delay in the patient's condition. In terms of treatment, the management of LCA typically involves surgical resection, with the approach tailored to the specific circumstances of the patient. Due to growth issues that have resulted in limited abdominal space and an enlarged spleen, laparoscopic surgery was not feasible, and open surgery needed to be considered. Although open surgery may carry relatively higher risks, it is sometimes necessary under such circumstances.

In conclusion, we reported a 14-year-old boy with pancytopenia and LCA of a huge spleen, with no recurrence within 8 months of follow-up (Supplementary Figure S2). Further details regarding the patient's postoperative recovery conditions are presented in Supplementary Table S4. However, due to this being a single case with a short follow-up duration, the relationship between pancytopenia and LCA needs further study.

## Data Availability

The original contributions presented in the study are included in the article/Supplementary Material, further inquiries can be directed to the corresponding author.
