# Supplementary material for: Littoral cell angioma of a huge spleen with peripheral blood pancytopenia in a 14-year-old boy: a case report and review of the literature
Source: Front Pediatr. 2024 Oct 31;12:1473930. doi: 10.3389/fped.2024.1473930 (PMC11560461; doi:10.3389/fped.2024.1473930)
Supplement: Supplementary file 1 [file Datasheet1.pdf]

Supplementary Table 1 Preoperative conditions of the patient.

Preoperative conditions	value
**Age (year)**	14
**Sex**	male
**Temperature (℃)**	36.3
**Pulse (bpm)**	100
**Respiration Rate**	20
**Blood pressure (mmHg)**	100/71
**Infectious disease**	No
**Blood transfusion history**	No
**Previous illnesses**	
Growth delay	
Moderate protein-energy malnutrition	
**Operation history**	No
**Admission history**	No
**Family medical history**	No
**Height and weight**	
Height (cm)	148.0
Weight (kg)	35.0
**BMI (kg/m^2)**	15.98
**Blood cell test**	
White blood cells (range 4.10-11.00× 10^9/L)	2.27× 10^9/L
Neutrophils (range 1.80-8.30× 10^9/L)	0.88× 10^9/L
Lymphocytes (range 1.20-3.80× 10^9/L)	1.18× 10^9/L
Monocytes (range 0.14-0.74× 10^9/L)	0.17× 10^9/L
Eosinophils (range 0.00-0.68× 10^9/L)	0.03× 10^9/L
Basophils (range 0.00-0.07× 10^9/L)	0.01× 10^9/L
Red blood cells (range 4.50-5.90× 10^{12}/L)	3.93× 10^{12}/L
Platelets (range 150-407× 10^9/L)	40× 10^9/L

Hemoglobin (range 129-172 g/L)	109 g/L
**Tumor markers test**	
Carcinoembryonic antigen (range 0.00-3.40 ng/mL)	<0.30 ng/mL
Carbohydrate antigen 125 (range 0.00-35.00 u/mL)	9.26 u/mL
Carbohydrate antigen 199 (range 0.00-27.00 u/mL)	2.88 u/mL
Alpha-fetoprotein (range 0.00-7.00 ng/mL)	1.55 ng/mL
**Spleen size**	
Computed tomography (length×width)	21.9 cm×9.1 cm
Abdominal ultrasonographic (length×width)	19.1 cm×8.5 cm
**Spleen Index**	83 cm^2
**Tumor size**	
Computed tomography (length×width)	9.3 cm×7.8 cm
Abdominal ultrasonographic (length×width)	7.4 cm×6.8 cm

Supplementary Table 2 Intraoperative conditions of the patient.

Intraoperative conditions	value
**The operation time (min)**	240
**The intraoperative transfusion (ml)**	
Autologous blood transfusion	300
**The intraoperative blood loss (ml)**	50
**The number of lymph nodes dissected**	
Splenic lymph nodes	8
**Intraoperative complications**	No

Supplementary Table 3 Clinical data of children with LCA.

Cases	Age (yr)	Sex	Clinical presentation	Anemia	Decreased PLT	Pancytopenia	Imaging results	Tumor types	Biomarkers	Treatment and prognosis
1[1]	1	Famel	Splenomegaly, fever	Yes	Yes	No	MRI: high density	Multiple	Factor VIII, CD31, CD34, CD68^{++++}	Splenectomy, No recurrence
2[2]	2	Male	Splenomegaly	Yes	Yes	No	CT: low-density shadows, MRI: mild progressive contrast enhancement, Ultrasound: low-density shadows	Multiple	Unknown	Splenectomy
3[3]	11	Famel	Abdominal symptoms	No	No	No	MRI: T1 hypointense signal, T2 hyperintense signal	Solitary	CD31, CD68, CD34^{++-}, CD8^{-}, Ki67 (2%)	Splenectomy
4[4]	12	Male	Splenomegaly	Unknown	Unknown	Unknown	Ultrasound: low-density	Multiple	Unknown	Splenectomy
5[5]	15	Male	Abdominal symptoms	No	No	No	Enhanced CT: high density in early arterial phase, low density in portal venous phase	Solitary	Unknown	Splenectomy
6[6]	26 days	Male	Vomiting, Abdominal symptoms	No	Yes	No	CT: low echo MRI: T1 and T2 hypointense signal	Multiple	ERG, CD68 and CD21, CD8^{+++-}, CD34^{-},	Conservative, No recurrence
7[7]	2	Male	Purpura, Splenomegaly	Yes	No	No	Unknown	Unknown	Unknown	Splenectomy, No recurrence

8[8]	11	Male	Splenomegaly, Abdominal symptoms, Anemia	Yes	Yes	No	Ultrasound: low echoes	Multiple	CD68, CD31, $CD34^{+++}$	Splenectomy
9[9]	9	Famel	Abdominal symptoms	Unknown	Unknown	Unknown	CT: mixed density of the spleen, central patchy high-density Ultrasound: heterogeneous echoes	Solitary	CD31, $CD34^{+-}$, ERG, EMA, Vim^{++-}, $Ki\text{-}67^{+}$	Splenectomy
10[10]	3	Famel	Asymptomatic	Yes	No	No	CT: low-density Ultrasound: slightly stronger echoic	Multiple	Vimentin/CD31, ERG, CD34, CD68, CD163, $CD21^{+++++++-}$, $Ki67^{+}$	Splenectomy, No recurrence
11[11]	9	Male	Abdominal symptoms, Splenomegaly	Unknown	Unknown	Unknown	MRI: T1 and T2 slightly prolonged/equal	Solitary	CD31, $CD34^{++}$, Ki-67, Vimentin, $CD68^{+++}$	Unknown
12[12]	7	Famel	Asymptomatic	No	No	No	MRI: slightly long T1, isointense T2	Solitary	CD31, CD34, $Ki67^{+++}$	Splenectomy, No recurrence
Our case	14	Male	Abdominal symptoms	Yes	Yes	Yes	CT: low-density, Enhanced CT: mixed density of the spleen, central patchy slightly high-density Ultrasound: heterogeneous echoes	Multiple	CD31. CD34, $CD68^{+++}$, Ki67(1%), $CD8^{-}$,	Splenectomy, No recurrence

Note: CT: computed Tomography; MRI: magnetic resonance imaging;

[1]Antón-Pacheo J, Ayuso RF, Cano I, et al. Splenic littoral cell angioma in an infant. Journal of pediatric surgery, 2000, 35(3): 508-509
[2]Ertan G, Tekes A, Mitchell S, et al. Pediatric littoral cell angioma of the spleen: multimodality imaging including diffusion-weighted imaging. Pediatric radiology, 2009, 39: 1105-1109
[3]Bedir R, Şehitoğlu İ, Calapoğlu AS, et al. A rare case of splenic littoral cell angioma in a child. Journal of Laboratory Physicians, 2014, 6(02): 117-120
[4]Forest F, Duband S, Clemenson A, et al. Traumatic subcapsular splenic hematoma revealing littoral cell angioma and Gaucher's disease. Annals of hematology, 2010, 89: 1061-1062
[5]Li Y, Wang X, Cai Y, et al. Laparoscopic central splenectomy for littoral cell angioma. Journal of Gastrointestinal Surgery, 2021, 25: 576-577
[6]Gakenheimer-Smith L, Mohlman J, VandenHeuvel K, et al. A novel presentation of littoral cell angioma and lymphatic malformations in a neonate. Pediatrics, 2018, 141(Supplement_5): S520-S525
[7]Jasani M, Shah A and Shah A. Littoral cell angioma: A rare cause of pediatric thrombocytopenia. Journal of Indian Association of Pediatric Surgeons, 2018, 23(3): 156-157
[8]Anbardar MH, Kumar PV and Forootan HR. Littoral cell angioma of the spleen: Cytological findings and review of the literature. Journal of Cytology, 2017, 34(2): 121-124
[9]Wang C, Tian Y, Cai W, et al. Littoral Cell Angioma of Spleen Complicated With Hemorrhage–2019 Film Reading Window (9). Anhui Yixue, 2019, 40(9): 1076-1077
[10]Mou Y, Yang L, Wang J, et al. Case report and literature review: Asymptomatic littoral cell angioma in a 3-year-old girl. Frontiers in Pediatrics, 2024, 12: 1383015
[11]Lai B, Zhong Q, Peng J. Case 149 answer of diagnose please: rare single littoral cell angioma of spleen in children. Radiol Pract. (2021) 36(3):419–420
[12]Wu R. Littoral Cell Angioma of the Spleen: Report of 10 Cases and Literature Review. Jinan: Shandong University; (2020).

Supplementary Table 4 Postoperative recovery of the patient.

Postoperative recovery	value
**Postoperative early complications**	
Fever	
**Postoperative late complications**	No
**Time to soft diet (d)**	1
**Hospital day after surgery (d)**	8
**Pain score at postoperative day**	2
**Blood cell test (after 1 month)**	
White blood cells (range 4.10-11.00× 10^9/L)	7.14× 10^9/L
Neutrophils (range 1.80-8.30× 10^9/L)	3.44× 10^9/L
Lymphocytes (range 1.20-3.80× 10^9/L)	1.36× 10^9/L
Monocytes (range 0.14-0.74× 10^9/L)	0.98× 10^9/L
Eosinophils (range 0.00-0.68× 10^9/L)	1.19× 10^9/L
Basophils (range 0.00-0.07× 10^9/L)	0.17× 10^9/L
Red blood cells (range 4.50-5.90× 10^{12}/L)	5.13× 10^{12}/L
Platelets (range 150-407× 10^9/L)	545× 10^9/L
Hemoglobin (range 129-172 g/L)	138 g/L
**Blood cell test (after 3 months)**	
White blood cells (range 4.10-11.00× 10^9/L)	11.1× 10^9/L
Neutrophils (range 1.80-8.30× 10^9/L)	4.39× 10^9/L
Lymphocytes (range 1.20-3.80× 10^9/L)	5.14× 10^9/L
Monocytes (range 0.14-0.74× 10^9/L)	0.84× 10^9/L
Eosinophils (range 0.00-0.68× 10^9/L)	0.66× 10^9/L
Basophils (range 0.00-0.07× 10^9/L)	0.08× 10^9/L
Red blood cells (range 4.50-5.90× 10^{12}/L)	5.20× 10^{12}/L
Platelets (range 150-407× 10^9/L)	185× 10^9/L
Hemoglobin (range 129-172 g/L)	139 g/L
**Weight (kg)**	
After 1 month	34.3
After 3 months	38.2
After 8 months	41.6
**Height (cm)**	
After 1 month	148.0
After 3 months	149.2

After 8 months	151.4
**BMI (kg/m^2)**	
After 1 month	15.66
After 3 months	17.16
After 8 months	18.15

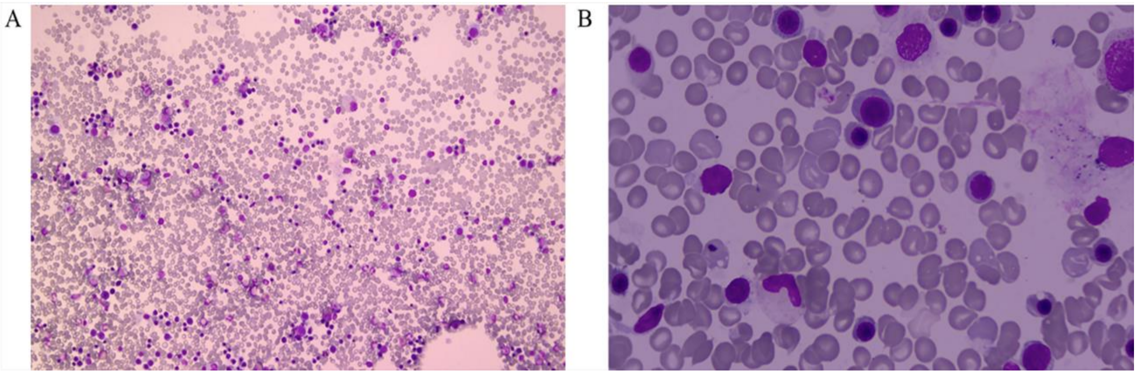

Supplementary Figure 1 Blood system check (A) The blood smear from the peripheral blood showed that blood cell morphology was normal. (HE ×100). (B) The blood smear from the bone marrow aspiration showed that megakaryocytic series and erythrocytic series increased but granulocytic series decreased. (HE × 100).

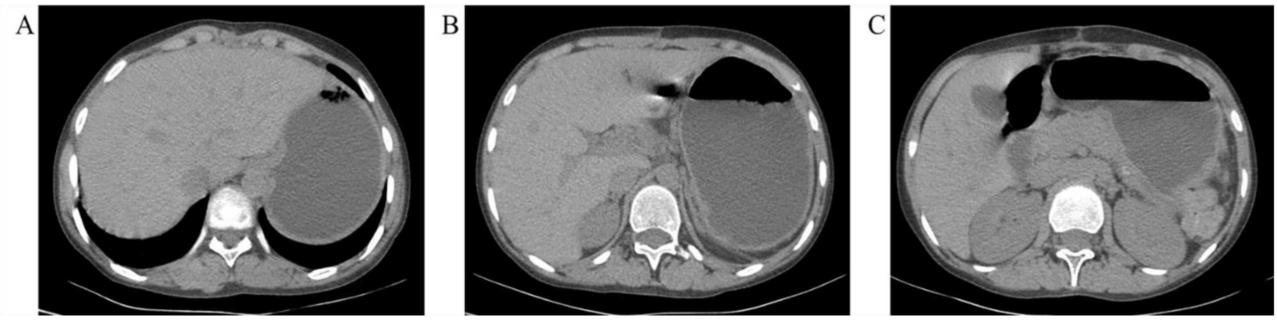

Supplementary Figure 2 Postoperative CT (A,B,C) shown that no abnormal lesions were found in the surgical area.
